# Supplementary material for: FERN – a Java framework for stochastic simulation and evaluation of reaction networks
Source: BMC Bioinformatics. 2008 Aug 29;9:356. doi: 10.1186/1471-2105-9-356 (PMC2553347; doi:10.1186/1471-2105-9-356)
Supplement: Additional file 1 — FERN distribution, Version 1.3. This archive contains the FERN source code and binaries as well as documentation and example models in FernML and SBML. [file 1471-2105-9-356-S1.zip › fern/doc/javadoc/fern/network/package-use.html]

Uses of Package fern.network


---


|  |  |  |
| --- | --- | --- |
| **Overview** \| **Package** \| Class \| **Use** \| **Tree** \| **Deprecated** \| **Index** \| **Help** | |  |
| PREV   NEXT | **FRAMES**    **NO FRAMES**     **All Classes** |  |


---


## **Uses of Package fern.network**

| Packages that use fern.network | |
| --- | --- |
| **fern.analysis** | Provides classes and algorithms for analysing networks like ShortestPath, AutocatalyticDetection. |
| **fern.benchmark** | Provides classes for benchmarking simulations (time benchmarks, histogram distance calculation). |
| **fern.cellDesigner** |  |
| **fern.cellDesigner.ui** |  |
| **fern.cytoscape** | Provides the classes for the cytoscape plugin. |
| **fern.example** | Contains examples and demonstrations of the framework. |
| **fern.network** | Provides general classes and interfaces for storing network data. |
| **fern.network.creation** | Provides classes for the evolution of networks. |
| **fern.network.fernml** | Provides the classes for parsing and using FernML based networks. |
| **fern.network.modification** | Provides classes for modifications of networks. |
| **fern.network.sbml** | Provides the classes for parsing and using sbml based networks. |
| **fern.simulation** | Provides algorithms for simulating reaction network as well as methods for observer certain aspects of a simulation. |
| **fern.simulation.algorithm** | Provides algorithms for stochastic simultion of reaction networks. |
| **fern.tools** | Provides common used tools. |

Classes in fern.network used by fern.analysis

****Network****
  
          The central interface of the whole framework.

Classes in fern.network used by fern.benchmark

****Network****
  
          The central interface of the whole framework.

Classes in fern.network used by fern.cellDesigner

****AbstractNetworkImpl****
  
          Base implementation for the `Network` interface.

****AmountManager****
  
          The `AmountManager` is one of the most important connections between a
`Network` and a `Simulator`.

****ComplexDependenciesPropensityCalculator****
  
          In a SBML network, the propensities of reaction are calculated by using the
kineticLaw tag, which contains a MathML expression.

****Network****
  
          The central interface of the whole framework.

****PropensityCalculator****
  
          A `PropensityCalculator` is the way, a `Network` tells a `Simulator`
how to calculate propensities for the reactions.

Classes in fern.network used by fern.cellDesigner.ui

****Network****
  
          The central interface of the whole framework.

Classes in fern.network used by fern.cytoscape

****AbstractNetworkImpl****
  
          Base implementation for the `Network` interface.

****AnnotationManager****
  
          AnnotationManager objects are the places, where static (or quasi-static) properties
of a network are stored.

****Network****
  
          The central interface of the whole framework.

Classes in fern.network used by fern.example

****FeatureNotSupportedException****

Classes in fern.network used by fern.network

****AbstractKineticConstantPropensityCalculator****
  
          Base implementation of a `PropensityCalculator`.

****AmountManager****
  
          The `AmountManager` is one of the most important connections between a
`Network` and a `Simulator`.

****AnnotationManager****
  
          AnnotationManager objects are the places, where static (or quasi-static) properties
of a network are stored.

****FeatureNotSupportedException****

****Network****
  
          The central interface of the whole framework.

****PropensityCalculator****
  
          A `PropensityCalculator` is the way, a `Network` tells a `Simulator`
how to calculate propensities for the reactions.

Classes in fern.network used by fern.network.creation

****AbstractNetworkImpl****
  
          Base implementation for the `Network` interface.

****Network****
  
          The central interface of the whole framework.

****PropensityCalculator****
  
          A `PropensityCalculator` is the way, a `Network` tells a `Simulator`
how to calculate propensities for the reactions.

Classes in fern.network used by fern.network.fernml

****AbstractNetworkImpl****
  
          Base implementation for the `Network` interface.

****AnnotationManager****
  
          AnnotationManager objects are the places, where static (or quasi-static) properties
of a network are stored.

****Network****
  
          The central interface of the whole framework.

Classes in fern.network used by fern.network.modification

****AmountManager****
  
          The `AmountManager` is one of the most important connections between a
`Network` and a `Simulator`.

****AnnotationManager****
  
          AnnotationManager objects are the places, where static (or quasi-static) properties
of a network are stored.

****Network****
  
          The central interface of the whole framework.

****PropensityCalculator****
  
          A `PropensityCalculator` is the way, a `Network` tells a `Simulator`
how to calculate propensities for the reactions.

Classes in fern.network used by fern.network.sbml

****AbstractNetworkImpl****
  
          Base implementation for the `Network` interface.

****AmountManager****
  
          The `AmountManager` is one of the most important connections between a
`Network` and a `Simulator`.

****ComplexDependenciesPropensityCalculator****
  
          In a SBML network, the propensities of reaction are calculated by using the
kineticLaw tag, which contains a MathML expression.

****FeatureNotSupportedException****

****Network****
  
          The central interface of the whole framework.

****PropensityCalculator****
  
          A `PropensityCalculator` is the way, a `Network` tells a `Simulator`
how to calculate propensities for the reactions.

Classes in fern.network used by fern.simulation

****AmountManager****
  
          The `AmountManager` is one of the most important connections between a
`Network` and a `Simulator`.

****Network****
  
          The central interface of the whole framework.

****PropensityCalculator****
  
          A `PropensityCalculator` is the way, a `Network` tells a `Simulator`
how to calculate propensities for the reactions.

Classes in fern.network used by fern.simulation.algorithm

****Network****
  
          The central interface of the whole framework.

Classes in fern.network used by fern.tools

****FeatureNotSupportedException****

****Network****
  
          The central interface of the whole framework.

---


|  |  |  |
| --- | --- | --- |
| **Overview** \| **Package** \| Class \| **Use** \| **Tree** \| **Deprecated** \| **Index** \| **Help** | |  |
| PREV   NEXT | **FRAMES**    **NO FRAMES**     **All Classes** |  |


---
